# Supplementary material for: Advances on mechanical designs for assistive ankle-foot orthoses
Source: Front Bioeng Biotechnol. 2023 Jul 7;11:1188685. doi: 10.3389/fbioe.2023.1188685 (PMC10361304; doi:10.3389/fbioe.2023.1188685)
Supplement: Supplementary file 1 [file DataSheet1.pdf]

Supplementary Material

1 SUPPLEMENTARY TABLES

Table S1. Complement of Table 1 of the main manuscript. The publications are classified depending on the type of assistance and purpose (empowerment or rehabilitation) across passive, quasi-passive and active devices. Note than some devices are intended to support both impaired and healthy users.

			Type of assistance					
			Drop-foot	Foot-slap	Push-off	Full gait	Drop-foot + foot-slap	Drop-foot + push-off
Passive	Impaired assist.	**11**	[1, 2]	[3, 4]	[5, 6, 7]	[8, 9, 10, 11, 12, 13, 14]		
	Healthy assist.	**3**			[15, 16, 17, 18]			
	Both assist.	**2**			[19, 20]			
	Rehabilitation							
	TOTAL	**16**	3	1	8	4		
Quasi-passive	Impaired assist.	**2**	[21]				[22]	
	Healthy assist.	**3**			[23, 24, 25]			
	Both assist.	**1**			[26]			
	Rehabilitation							
	TOTAL	**6**	1		4		1	
Active	Impaired assist.	**19**	[27, 28]		[29, 30, 31, 32, 33, 34]	[35, 36, 37, 38, 39, 40, 41, 42, 43, 44, 45, 46, 47, 48, 49, 50, 51, 52, 53, 54]	[55]	[56, 57, 58, 59, 60, 61, 62, 63, 64]
	Healthy assist.	**10**			[65, 66, 67, 68, 69, 70, 71, 72, 73, 74, 73, 75, 76, 77, 78, 79, 80, 81, 82, 83, 84, 85, 86, 87, 88, 89, 77]			
	Both assist.	**1**				[90, 91, 92]		
	Rehabilitation	**2**				[93, 94, 95]		
	TOTAL	**32**	2		15	10	1	4

Table S2: Summary of reviewed passive AAFOs and their effects when being tested in subjects.

Ref.	Assisted DOFs	Cluster	Weight (kg)	Max. ROM (deg)	Max. Assist. Spec.	Assist. Type	Subjects	Kinematics	EMG	Energy consumption
[19]	1/3	BWC	0.459	Full ROM	0.1-0.2 Nm/kg	Push-off	2 healthy	The exoskeleton adapts to changes in walking speeds	Reductions of 5-17% in average soleus activity comparing to no-exo condition	-
[8, 9, 10]	1/1	ADR	-	±15	0.4 Nm/kg	Full gait	15 children with CP	Observed reduction on knee flexion during stance similar to the one achieved by rigid AFOs. Worse trunk performance and gait instability when wearing the ADR-AAFO	-	No reduction
[11, 12]	1/1	ADR	-	±40	Adult ±68Nm; Child ±15.8 Nm	Full gait	10 children with CP	ADR produced better knee extension and push-off power than PLS-AFO. PLS produced more normal ankle motion, better walking and greater parent satisfaction	-	-

Table S2: Summary of reviewed passive AAFOs and their effects when being tested in subjects.

Ref.	Assisted DOFs	Cluster	Weight (kg)	Max. ROM (deg)	Max. Assist. Spec.	Assist. Type	Subjects	Kinematics	EMG	Energy consumption
[13]	1/1	ADR	-	±18	±30 Nm	Full gait	10 stroke survivors	Ankle and knee kinematics were affected by changes on the AAFO parameters (spring resistance, zero alignment…)	-	-
[1]	1/1	Spring-CAM	1.4	+25 / -20	-	Drop-foot	1 healthy	No changes	Reduced tibialis anterior activity during swing phase (43.9% of compensation)	-
[15]	1/1	BWC	0.7655	Full ROM	-	Push-off	5 healthy	The exo introduced inertia that affected the foot positioning and clearance. Shorter stride length. Drop foot occurred while wearing the device	Soleus activity reduced by 72%	-

Table S2: Summary of reviewed passive AAFOs and their effects when being tested in subjects.

Ref.	Assisted DOFs	Cluster	Weight (kg)	Max. ROM (deg)	Max. Assist. Spec.	Assist. Type	Subjects	Kinematics	EMG	Energy consumption
[16, 17]	1/1	AAC	0.408 - 0.503	Full ROM	0.37 Nm/kg	Push-off	9 healthy	The exo did not interfere with normal kinematics	Soleus activity decreased with increasing spring stiffness. Gastroc. activity decreased during early and mid-stance, but increased during late stance. Tibialis anterior activity increased during early and mid-stance, and ws unchanged during late stance	Reduction of the metabolic cost of walking by 7.2±2.6% for healthy human users
[14]	1/1	ADR	-	?/-20	-	Full gait	-	-	-	-

Table S2: Summary of reviewed passive AAFOs and their effects when being tested in subjects.

Ref.	Assisted DOFs	Cluster	Weight (kg)	Max. ROM (deg)	Max. Assist. Spec.	Assist. Type	Subjects	Kinematics	EMG	Energy consumption
[5]	1/1	Spring-CAM	0.435	Full ROM	-	Push-off	11 stroke survivors with hemiparesis	The device (AAFO-PCAM) facilitated the realization of the ankle plantarflexion power in the late-stance phase (compared to just AFO-P) because of dorsiflexion resistance, increasing the knee flexion angle during the swing phase	-	-
[20]	1/1	AAC	1.45	+45 / -20	115 Nm (technical tests)	Push-off	-	-	-	-
[6]	-	BWC	-	-	0.3 Nm/kg	Push-off	-	-	-	-
[2]	1/1	BWC	-	0 / Full dorsiflexion	4.07 Nm	Drop-foot	1 healthy	Prevented drop foot when the subject tried to simulate it. The steps were longer with the AAFO than without AAFO when simulating drop foot	-	-
[7]	1/1	BWC	-	Full ROM	-	Push-off	-	-	-	-

Table S2: Summary of reviewed passive AAFOs and their effects when being tested in subjects.

Ref.	Assisted DOFs	Cluster	Weight (kg)	Max. ROM (deg)	Max. Assist. Spec.	Assist. Type	Subjects	Kinematics	EMG	Energy consumption
[18]	1/1	AAC	1.51	Full ROM with compliant PAM	-	Push-off	5 healthy	ROM of both ankles was affected by wearing a unilateral device. The device minimally affected the walking speed as well as the angular ROM of the knee and hip joints	Activation of the calf muscles (Gas Med and Soleus) was reduced on the leg with the device	-
[3, 4]	1/1	Other	0.1 (only mechanism)	+18/-30	14 Nm	Foot-slap	17 stroke survivors	More ankle dorsiflexion in single stance and more peak power absorption in stance for the AAFO-OD compared to a rigid AFO group. No difference found in peak plantarfexion moment, ankle power generation, spatial or temporal parameters, ground reaction force, or shank-to-vertical angle between the two groups	-	-

BWC: body-weight dependent clutch; ADR: adjustable dynamic response; AAC: ankle-angle dependent clutch; Max. ROM: plantarflexion > 0; dorsiflexion < 0

Table S3: Summary of reviewed quasi-passive AAFOs and their effects when being tested in subjects.

Ref.	Assisted DOFs	Cluster	Weight (kg)	Max. ROM (deg)	Max. Assist. Spec.	Assist. Type	Subjects	Kinematics	EMG	Energy consumption
[26]	1/1	EC	0.754	?/-12	0.27 Nm/kg	Push-off	6 healthy	No changes	Level ground: reductions in SOL (6.8±1.4%) and GAS (6.6±1.0%). Increase in TA (4.4±0.8%)	Level ground: reductions of 6.4±1.3%; - Ramp up: reductions of 5.2±1.2% at 10% grade and of 4.1±1.1% at 15% grade; Ramp down: increase of 2.6±1.0% at 10% grade and by 2.4 ± 0.5% at 15% grade
[96]	1/3	EC	-	+29/-19	120 Nm	Push-off	-	-	-	-
[21]	1/1	Other	1.3	-	7.8 N	Drop-foot	-	-	-	-
[23]	1/1	Other	-	±30	7.37 Nm	Push-off	1 healthy	-	-	-
[22]	1/1	Other	0.819	+20/-?	1.88 Nm	Drop-foot and foot-slap	7 healthy		-	-
[24]	1/1	Other	1.75	-	362 Nm/rad	Push-off	1 healthy	-	Reductions of 26.48% for TA and 7.42% for SOL	-

Table S3: Summary of reviewed quasi-passive AAFOs and their effects when being tested in subjects.

Ref.	Assisted DOFs	Cluster	Weight (kg)	Max. ROM (deg)	Max. Assist. Spec.	Assist. Type	Subjects	Kinematics	EMG	Energy consumption
[25]	1/1	EC	1.09	± 10	-	Push-off	7 healthy	-	-	-

EC: electrically-controlled clutch; Max. ROM: plantarflexion > 0; dorsiflexion < 0

Table S4: Summary of reviewed active AAFOs and their effects when being tested in subjects.

Ref.	Assisted DOFs	Cluster	Weight (kg)	Max. ROM (deg)	Max. Assist. Spec.	Assist. Type	Control	Subjects	Kinematics	EMG	Energy consumption
[27]	1/1	SEA	2.6	-	-	Drop-foot	Impedance controller. Impedance level depends on gait state (swing/stance)	2 patients with drop-foot	Improvement of swing kinematics and reduction of foot-slap	-	-
[55]	1/1	SEA	2.8	+21.5/-12	-	Drop-foot and foot-slap	The SEA is shortened or lengthened to induce dorsiflexion or plantarflexion. Torque direction according to the gait state	3 hemiparetic patients	Improvement of spatio-temporal parameters by preventing drop-foot	-	-
[35, 36, 37]	2/3	SEA	3.6	+45/-25	23Nm	Full gait	Impedance controller - Variable impedance and assistive torque profiles depending on biomechanical models and current gait state	> 20 patients	Reduced drop-foot	-	-

Table S4: Summary of reviewed active AAFOs and their effects when being tested in subjects.

Ref.	Assisted DOFs	Cluster	Weight (kg)	Max. ROM (deg)	Max. Assist. Spec.	Assist. Type	Control	Subjects	Kinematics	EMG	Energy consumption
[38]	1/1	RT	0.869	-	20Nm	Full gait	Position control	-	-	-	-
[29, 30]	1/1	SEA	0.95 (only actuator)	-	110W	Push-off	Position control for spring compression: Assistive profile triggered by heel-strike and scaled to previous step duration [29]; Storage/release of energy depending on the gait state [30]	3 stroke survivors	[30] gait features improvements	-	-
[28]	1/1	RT	2.6	+45/-20	9.8Nm	Drop-foot	Torque control. Finite state machine to detect swing/stance. Free motion during stance, assistive torque during swing	5 hemiparetic patients	Ankle dorsiflexion peak improved	-	-
[65, 66, 67]	1/1	Cable driven	1.06	-	2W/Kg	Push-off	Torque control. Assistive profile triggered by heel-strike	> 20 healthy	The provided mechanical power replaces power at biological joints	-	Metabolic cost reduced

Table S4: Summary of reviewed active AAFOs and their effects when being tested in subjects.

Ref.	Assisted DOFs	Cluster	Weight (kg)	Max. ROM (deg)	Max. Assist. Spec.	Assist. Type	Control	Subjects	Kinematics	EMG	Energy consumption
[68, 69, 70, 71]	1/1	Cable driven	1.5	-	30Nm	Push-off	Torque control. Assistive profile triggered by heel-strike (deep learning model for gait state estimation in [71])	> 20 healthy	[70] participants changed the manner in which they maintained stability while on a beam (changes in the normalized stride length and duration), although overall balance was not affected; [68] Individuals exhibited different sensitivity towards actuation timing	[69] 60% subjects reduced GM and 80% increased TA activity; muscular response highly varied between subjects	[71] 5.2% decrease in metabolic expenditure
[93, 94]	1/1	RT	1.9	-	9Nm	Full gait	Non-regulated torque control. [93] Footswitch signals trigger the torque application;[94] Continuous phase estimation based on cross-correlation with a learned model	-	-	-	-

Table S4: Summary of reviewed active AAFOs and their effects when being tested in subjects.

Ref.	Assisted DOFs	Cluster	Weight (kg)	Max. ROM (deg)	Max. Assist. Spec.	Assist. Type	Control	Subjects	Kinematics	EMG	Energy consumption
[39, 40, 41]	1/1	RT	1	+30/-20	16.7Nm	Full gait	Torque control. State machine to detect gait states and execute different motor profiles	3-14 stroke survivors	Drop-foot and foot-slap reduced, improved gait pattern and speed	-	-
[56]	1/1	Cable driven	1	-	7.3Nm	Drop-foot and push-off	Torque control. Torque/force reference triggered by gait events	5 healthy	Confirmed feasibility for foot-drop prevention and propulsion assistance	Confirmed feasibility for drop-foot prevention and propulsion assistance	-

Table S4: Summary of reviewed active AAFOs and their effects when being tested in subjects.

Ref.	Assisted DOFs	Cluster	Weight (kg)	Max. ROM (deg)	Max. Assist. Spec.	Assist. Type	Control	Subjects	Kinematics	EMG	Energy consumption
[42, 43, 44, 45, 46, 47, 48, 49, 50, 51]	1/1	Cable driven	1.85-2.2	-	20Nm	Full gait	Torque control. [42] Assistive torque profile triggered by gait events detection; [43, 46, 50, 51] assistance proportional to ankle moment during stance; [44, 45, 47] assistance proportional to ankle moment; [48] resistance proportional to ankle moment	> 20 patients with CP	Improved ankle plantarflexion and knee extension, producing a more efficient gait pattern, improved 6-min walking test performance, more symmetrical gait pattern with bilateral assistance	Main reduction on soleus activity	Metabolic cost reduced
[57, 58]	1/1	Cable driven	2.8	-	4.5Nm	Drop-foot and push-off	Torque control. Gait phases detected by inertial sensor placed on the foot that triggers the assistance	10 stroke survivors	Biomechanical improvements in 70% patients	-	-

Table S4: Summary of reviewed active AAFOs and their effects when being tested in subjects.

Ref.	Assisted DOFs	Cluster	Weight (kg)	Max. ROM (deg)	Max. Assist. Spec.	Assist. Type	Control	Subjects	Kinematics	EMG	Energy consumption
[72, 73, 74]	1/1	Cable driven	0.88	+30/-20	119Nm	Push-off	Torque or work control. Torque profile triggered by heel strike and scaled to last step duration	> 20 healthy	-	Reduced muscular activity and effect on muscular coordination	Metabolic cost reduced with work assistance but increased with torque assistance
[73, 75, 76, 77]	1/1	Cable driven	0.88	+30/-20	121Nm	Push-off	Torque or work control. Torque profile triggered by heel strike and scaled to last step duration	> 20 healthy	-	Reduced soleus activity	Human-in-the-loop (EMG or torque optimized) reduced metabolic cost
[97, 98, 99, 100, 101]	1/1	PAM	0.89	?/-15	-	Push-off	Work control. Torque profile triggered by heel strike and scaled to last step duration	> 20 healthy	Walking performance increased (subjects were able to carry more weight)	Reduced leg muscular activity	Metabolic cost reduced

Table S4: Summary of reviewed active AAFOs and their effects when being tested in subjects.

Ref.	Assisted DOFs	Cluster	Weight (kg)	Max. ROM (deg)	Max. Assist. Spec.	Assist. Type	Control	Subjects	Kinematics	EMG	Energy consumption
[102]	1/1	RT	-	-	-	Full gait	Position tracking by adaptive proxy-based sliding mode control. Trajectory generation depending on gait phase estimation based on time between key-events	Technically validated	-	-	-
[59, 60, 61, 62, 63]	1/3	Cable driven	3.8	Full ROM	300N	Drop-foot and push-off	[59, 60, 61] cable position control; [62] iterative Force-based cable trajectory tracking; [63] offline human-in-the-loop optimized force profiles tracked by an admittance control	$>$ 20 stroke survivors	Improved gait symmetry, reduced hip hiking and circumduction	-	Metabolic cost reduced

Table S4: Summary of reviewed active AAFOs and their effects when being tested in subjects.

Ref.	Assisted DOFs	Cluster	Weight (kg)	Max. ROM (deg)	Max. Assist. Spec.	Assist. Type	Control	Subjects	Kinematics	EMG	Energy consumption
[78]	1/1	Cable driven	0.348	Full ROM	30 Nm	Push-off	Open-loop torque control. Gait phase estimation from the duration of previous steps. Plantar-flexion torque profile triggered at certain phase	1 healthy subject	-	Gastrocnemius activity decreased	-
[79, 80, 81, 82]	1/1	SEA	1.5	-	100N	Push-off	Torque control tracking a spring simulated torque profile; [79] Gait phase estimated by an AO based on the foot pressure; [80, 81] Central Pattern Generator based on floor contact events	[79] 4 healthy subjects; [81] 4 incomplete spinal cord injury patients during 10-session training	Improved gait speed and endurance during assisted walking	Reduced muscle activity	[79] No difference in metabolic cost; [80] reduced metabolic cost of walking

Table S4: Summary of reviewed active AAFOs and their effects when being tested in subjects.

Ref.	Assisted DOFs	Cluster	Weight (kg)	Max. ROM (deg)	Max. Assist. Spec.	Assist. Type	Control	Subjects	Kinematics	EMG	Energy consumption
[83, 84, 85, 86]	1/1	PAM	1.2	-	60Nm	Push-off	Plantarflexor torque control. [83] proportional myoelectric control from soleus EMG; [84] Proportional myoelectric control from Medial Gastrocnemius EMG;[85] proportional soleus myoelectric control vs footswitch on/off control;[86] Proportional myoelectric control from soleus EMG with fixed gain	> 20 healthy	Kinematics closer to normal with myoelectric control. Differences in kinematic patterns but similar ankle torque pattern with and without exoskeleton.	Main reduction on soleus activity	-

Table S4: Summary of reviewed active AAFOs and their effects when being tested in subjects.

Ref.	Assisted DOFs	Cluster	Weight (kg)	Max. ROM (deg)	Max. Assist. Spec.	Assist. Type	Control	Subjects	Kinematics	EMG	Energy consumption
[87, 88, 89]	1/1	PAM	1.2	-	60Nm	Push-off	Plantarflexor torque control. [87] proportional myoelectric control from soleus EMG with fixed gain; [88] proportional myoelectric control from soleus EMG with adaptive gain; [89] proportional myoelectric control vs torque profile triggered by heel strikes	> 20 healthy	Similar to normal ankle pattern; reduced ankle power	Reduced soleus activity	Metabolic cost reduced
[31]	1/1	PAM	0.53	-	-	Push-off	Plantarflexor torque control. Proportional myoelectric propulsion from soleus EMG	5 stroke survivors	Increased paretic plantarflexor torque	-	-

Table S4: Summary of reviewed active AAFOs and their effects when being tested in subjects.

Ref.	Assisted DOFs	Cluster	Weight (kg)	Max. ROM (deg)	Max. Assist. Spec.	Assist. Type	Control	Subjects	Kinematics	EMG	Energy consumption
[32]	1/1	Cable driven	-	-	-	Push-off	Force control. Proportional myoelectric propulsion from soleus EMG increased by gait velocity	6 stroke survivors	Increased paretic plantarflexor torque	-	-
[90, 91, 92]	1/2	SEA	3.8	-	102Nm	Full gait	Torque controller. [92] neuromuscular controller; [90] balance recovering controller; [91] neuromechanical models to estimate biological ankle joint torques in real-time from measured EMG and joint angles	3 SCI subjects; 16 healthy	SCI patients achieved healthy-like kinematic patterns, balance improvements, reduced biological torques	Reduced muscular activity (soleus and medial gastrocnemius for recovering balance)	-

Table S4: Summary of reviewed active AAFOs and their effects when being tested in subjects.

Ref.	Assisted DOFs	Cluster	Weight (kg)	Max. ROM (deg)	Max. Assist. Spec.	Assist. Type	Control	Subjects	Kinematics	EMG	Energy consumption
[33, 34]	1/1	Cable driven	0.95-1.4	-	50Nm	Push-off	[33] Gait phase estimation to generate torque profile; [34] Study about performance of nine different torque profiles	3-5 healthy subjects	Exoskeleton assistance changed maximum ankle dorsiflexion and plantarflexion angle and reduced biological ankle moment	Soleus activity decreased	Metabolic cost reduced
[52, 53]	1/1	RT	1.56	-	30Nm	Full gait	Torque controller. Torque profile triggered by heel strike and scaled to previous steps duration	5 stroke survivors	Drop-foot correction and reduced hip compensatory movements	-	-
[77]	1/1	Cable driven	1.2	+55/?	54Nm	Push-off	Speed-adaptive torque controller optimized by AI	10 healthy subjects	Increased gait speed	-	Metabolic cost reduced
[103]	2/2	PAM	2.14	-	-	Push-off	Force/Position control	7 healthy subjects	Increase balance of inversion/eversion of the ankle	The EMG rate decreased by 17.1% ± 11.3% by PAM1	-

Table S4: Summary of reviewed active AAFOs and their effects when being tested in subjects.

Ref.	Assisted DOFs	Cluster	Weight (kg)	Max. ROM (deg)	Max. Assist. Spec.	Assist. Type	Control	Subjects	Kinematics	EMG	Energy consumption
[64]	1/2	RT	2.1	+50/-20	12Nm	Drop-foot and push-off	Impedance control dependent from gait state	1 healthy subject	Increased plantar and dorsiflexion are clearly shown	-	-
[95]	2/3	RT	1.355	+50/-20	21Nm	Full Gait	Position control	1 healthy subjects		Decrease of tibialis anterior and gastrocnemius peak EMG	
[54]	1/1	SEA	3.05	+40/-20	Driving 22.6Nm Braking 41.9 Nm	Full gait	Impedance control dependent from gait state	8 healthy subjects	-	Reduction of tibialis anterior and soleus EMG	-

RT: rigid transmission; SEA: series elastic actuator; PAM: pneumatic artificial muscle; Max. ROM: plantarflexion > 0; dorsiflexion < 0

REFERENCES

[1] Karen Rodriguez, Jurriaan De Groot, Frank Baas, Marjon Stijntjes, Frans Van Der Helm, Herman Van Der Kooij, and Winfred Mugge. Passive ankle joint stiffness compensation by a novel ankle-foot-orthosis. In *IEEE International Conference on Biomedical Robotics and Biomechatronics (BioRob)*, 2018.

[2] Hiroaki Hirai, Ryuta Ozawa, Satoru Goto, Hiroki Fujigaya, Shodo Yamasaki, Yasuhiko Hatanaka, and Sadao Kawamura. Development of an ankle-foot orthosis with a pneumatic passive element. In *IEEE International Workshop on Robot and Human Interactive Communication*, pages 220–225, 2006.

[3] S. Yamamoto, A. Hagiwara, T. Mizobe, O. Yokoyama, and T. Yasui. Development of an ankle-foot orthosis with an oil damper. *Prosthetics and Orthotics International*, 29(3):209–219, 2005.

[4] S Yamamoto, N Motojima, Y Kobayashi, Y Osada, S Tanaka, and A Daryabor. Ankle-foot orthosis with an oil damper versus nonarticulated ankle-foot orthosis in the gait of patients with subacute stroke: a randomized controlled trial. *Journal of NeuroEngineering and Rehabilitation*, 19(1), 2022.

[5] Yusuke Sekiguchi, Dai Owaki, Keita Honda, Kenichiro Fukushi, Noriyoshi Hiroi, Takeo Nozaki, and Shin ichi Izumi. Ankle–foot orthosis with dorsiflexion resistance using spring-cam mechanism increases knee flexion in the swing phase during walking in stroke patients with hemiplegia. *Gait and Posture*, 81(July):27–32, 2020.

[6] Marleen van Hoorn, Edsko Hekman, Eduardo Rocon, Edwin van Asseldonk, and Cristina Bayón. Technical validation of a body-weight controlled clutch for ankle-foot orthoses of children with cerebral palsy. *Proceedings - XLIII Jornadas de Automática, 2022, Logroño (La Rioja)*, pages 165–170, 2022.

[7] Lili Liu, Wenhao Wei, Kai Zheng, Yanan Diao, Zhuo Wang, Guanglin Li, Senior Member, and Guoru Zhao. Design of an Unpowered Ankle-Foot Exoskeleton Used for Walking Assistance. In *43rd Annual International Conference of the IEEE Engineering in Medicine & Biology Society (EMBC)IEEE Engineering in Medicine & Biology Society (EMBC)*, pages 4501–4504, 2021.

[8] Yvette L. Kerkum, Merel Anne Brehm, Annemieke I. Buizer, Josien C. Van Den Noort, Jules G. Becher, and Jaap Harlaar. Defining the mechanical properties of a spring-hinged ankle foot orthosis to assess its potential use in children with spastic cerebral palsy. *Journal of Applied Biomechanics*, 30(6):728–731, 2014.

[9] Yvette L Kerkum, Annemieke I Buizer, Josien C Van Den Noort, and Jules G Becher. The Effects of Varying Ankle Foot Orthosis Stiffness on Gait in Children with Spastic Cerebral Palsy Who Walk with Excessive Knee Flexion. *PLoS ONE*, 2015.

[10] P. Meyns, Y. L. Kerkum, M. A. Brehm, J. G. Becher, A. I. Buizer, and J. Harlaar. Ankle foot orthoses in cerebral palsy: Effects of ankle stiffness on trunk kinematics, gait stability and energy cost of walking. *European Journal of Paediatric Neurology*, 26:68–74, 2020.

[11] Tishya A.L. Wren, James W. Dryden, Nicole M. Mueske, Sandra W. Dennis, Bitte S. Healy, and Susan A. Rethlefsen. Comparison of 2 Orthotic Approaches in Children with Cerebral Palsy. *Pediatric Physical Therapy*, 27(3):218–226, 2015.

[12] Ultraflex Systems. Unprecedented Stability and Motion: Ultraflex's Adjustable Dynamic Response (ADR). Technical report, Ultraflex, 2009.

[13] Toshiki Kobayashi, Michael S. Orendurff, Grace Hunt, Fan Gao, Nicholas LeCursi, Lucas S. Lincoln, and K. Bo Foreman. The effects of alignment of an articulated ankle-foot orthosis on lower limb joint kinematics and kinetics during gait in individuals post-stroke. *Journal of Biomechanics*, 83:57–64, jan 2019.

[14]Ottobock. Nexgear Tango Ankle Joint. Technical report, Ottobock, 2018.

[15]Xiangyang Wang, Sheng Guo, Bojian Qu, Majun Song, and Haibo Qu. Design of a Passive Gait-based Ankle-foot Exoskeleton with Self-adaptive Capability. *Chinese Journal of Mechanical Engineering (English Edition)*, 33(1), 2020.

[16]M. Bruce Wiggin, Gregory S. Sawicki, and Steven H. Collins. An exoskeleton using controlled energy storage and release to aid ankle propulsion. In *IEEE International Conference on Rehabilitation Robotics*, 2011.

[17]Steven H. Collins, M. Bruce Wiggin, and Gregory S. Sawicki. Reducing the energy cost of human walking using an unpowered exoskeleton. *Nature*, 522(7555):212–215, 2015.

[18]Scott Pardoel and Marc Doumit. Development and testing of a passive ankle exoskeleton. *Biocybernetics and Biomedical Engineering*, 39(3):902–913, 2019.

[19]Matthew B. Yandell, Joshua R. Tacca, and Karl E. Zelik. Design of a Low Profile, Unpowered Ankle Exoskeleton That Fits Under Clothes: Overcoming Practical Barriers to Widespread Societal Adoption. *IEEE Transactions on Neural Systems and Rehabilitation Engineering*, 27(4):712–723, 2019.

[20]Justin Leclair, Scott Pardoel, Alexander Helal, and Marc Doumit. Development of an unpowered ankle exoskeleton for walking assist. *Disability and Rehabilitation: Assistive Technology*, 15(1):1–13, 2018.

[21]David P. Allen, Ryan Little, Joshua Laube, Jeremy Warren, Walter Voit, and Robert D. Gregg. Towards an ankle-foot orthosis powered by a dielectric elastomer actuator. *Mechatronics*, 76:102551, jun 2021.

[22]Takahiro Oba, Hideki Kadone, Modar Hassan, and Kenji Suzuki. Robotic Ankle–Foot Orthosis With a Variable Viscosity Link Using MR Fluid. *IEEE Transactions on mechatronics*, 24(2), 2019.

[23]Stuart Diller, Carmel Majidi, and Steven H. Collins. A lightweight, low-power electroadhesive clutch and spring for exoskeleton actuation. In *IEEE International Conference on Robotics and Automation*, 2016.

[24]Saurav Kumar, Matthew Richard Zwall, Edgar A. Bolívar-Nieto, Robert D. Gregg, and Nicholas Gans. Extremum Seeking Control for Stiffness Auto-Tuning of a Quasi-Passive Ankle Exoskeleton. *IEEE Robotics and Automation Letters*, 5(3), 2020.

[25]Miha Dezman, Jan Babic, and Adrej Gams. Qualitative Assessment of a Clutch-Actuated Ankle Exoskeleton. In *International Conference on Robotics in Alpe-Adria Danube Region*, 2018.

[26]Cunjin Wang, Lei Dai, Donghua Shen, Jiyuan Wu, Xingsong Wang, Mengqian Tian, Yunde Shi, and Chun Su. Design of an Ankle Exoskeleton That Recycles Energy to Assist Propulsion during Human Walking. *IEEE Transactions on Biomedical Engineering*, 69(3):1212–1224, 2022.

[27]J.A. Blaya and H Herr. Adaptive control of a variable-impedance ankle-foot orthosis to assist drop-foot gait. *IEEE Transactions on Neural Systems and Rehabilitation Engineering*, 12(1):24–31, mar 2004.

[28]Sangjoon Jonathan Kim, Youngjin Na, Dong Yeon Lee, Handdeut Chang, and Jung Kim. Pneumatic AFO Powered by a Miniature Custom Compressor for Drop Foot Correction. *IEEE Transactions on Neural Systems and Rehabilitation Engineering*, 28(8):1781–1789, 2020.

[29]A. Mehmet Oymagil, Joseph K. Hitt, Thomas Sugar, and Jennifer Fleeger. Control of a Regenerative Braking Powered Ankle Foot Orthosis. In *2007 IEEE 10th International Conference on Rehabilitation Robotics*, volume 00, pages 28–34. IEEE, jun 2007.

[30]Jeffrey Ward, Thomas Sugar, Alexander Boehler, John Standeven, and Jack R Engsberg. Stroke Survivors' Gait Adaptations to a Powered Ankle-Foot Orthosis. *ADVANCED ROBOTICS*, 25(15):1879–1901, 2011.

[31]K Z Takahashi, M D Lewek, and G S Sawicki. A neuromechanics-based powered ankle exoskeleton to assist walking post-stroke: A feasibility study. *Journal of NeuroEngineering and Rehabilitation*, 12(1), 2015.

[32]Emily M. McCain, Taylor J. M. Dick, Tracy N. Giest, Richard W. Nuckols, Michael D. Lewek, Katherine R. Saul, and Gregory S. Sawicki. Mechanics and energetics of post-stroke walking aided by a powered ankle exoskeleton with speed-adaptive myoelectric control. *Journal of NeuroEngineering and Rehabilitation*, 16(1):57, dec 2019.

[33]Jianyu Chen, Jianda Han, and Juanjuan Zhang. Design and Evaluation of a Mobile Ankle Exoskeleton With Switchable Actuation Configurations. *IEEE/ASME Transactions on Mechatronics*, pages 1–10, 2022.

[34]Wei Wang, Jianyu Chen, Jianquan Ding, Juanjuan Zhang, and Jingtai Liu. Improving Walking Economy With an Ankle Exoskeleton Prior to Human-in-the-Loop Optimization. *Frontiers in Neurorobotics*, 15(January):1–12, jan 2022.

[35]Anindo Roy, Hermano Igo Krebs, Dustin J. Williams, Christopher T. Bever, Forrester Larry W., Richard M. Macko, and Neville Hogan. Robot-Aided Neurorehabilitation: A Novel Robot for Ankle Rehabilitation. *IEEE Transactions on Robotics*, 25(3), 2009.

[36]Anindo Roy, Hermano I Krebs, Joseph E Barton, Richard F Macko, and Larry W Forrester. Anklebot-assisted locomotor training after stroke: A novel deficit-adjusted control approach. In *2013 IEEE International Conference on Robotics and Automation*, pages 2175–2182. IEEE, may 2013.

[37]Larry W Forrester, Anindo Roy, Charlene Hafer-Macko, Hermano I Krebs, and Richard F Macko. Task-specific ankle robotics gait training after stroke: a randomized pilot study. *Journal of NeuroEngineering and Rehabilitation*, 13(1):51, dec 2016.

[38]Hyundo Choi, Young Jin Park, Keehong Seo, Jusuk Lee, Sang Eui Lee, and Youngbo Shim. A Multifunctional Ankle Exoskeleton for Mobility Enhancement of Gait-Impaired Individuals and Seniors. *IEEE Robotics and Automation Letters*, 3(1):411–418, 2018.

[39]Ling-Fung Yeung, Cathy C Y Lau, Charles W K Lai, Yannie O Y Soo, Man-Lok Chan, and Raymond K Y Tong. Effects of wearable ankle robotics for stair and over-ground training on sub-acute stroke: a randomized controlled trial. *Journal of NeuroEngineering and Rehabilitation*, 18(1):19, dec 2021.

[40]Ling Fung Yeung, Corinna Ockenfeld, Man Kit Pang, Hon Wah Wai, Oi Yan Soo, Sheung Wai Li, and Kai Yu Tong. Randomized controlled trial of robot-assisted gait training with dorsiflexion assistance on chronic stroke patients wearing ankle-foot-orthosis. *Journal of NeuroEngineering and Rehabilitation*, 15(1):1–12, 2018.

[41]Ling-Fung Yeung, Corinna Ockenfeld, Man-Kit Pang, Hon-Wah Wai, Oi-Yan Soo, Sheung-Wai Li, and Kai-Yu Tong. Design of an exoskeleton ankle robot for robot-assisted gait training of stroke patients. In *2017 International Conference on Rehabilitation Robotics (ICORR)*, pages 211–215. IEEE, jul 2017.

[42]Zachary F. Lerner, Gian Maria Gasparri, Michael O. Bair, Jenny L. Lawson, Jason Luque, Taryn A. Harvey, and Andrea T. Lerner. An untethered ankle exoskeleton improves walking economy in a pilot study of individuals with cerebral palsy. *IEEE Transactions on Neural Systems and Rehabilitation Engineering*, 26(10):1985–1993, 2018.

[43]Zachary F. Lerner, Taryn A. Harvey, and Jennifer L. Lawson. A Battery-Powered Ankle Exoskeleton Improves Gait Mechanics in a Feasibility Study of Individuals with Cerebral Palsy. *Annals of Biomedical Engineering*, 47(6):1345–1356, 2019.

[44]Gian Maria Gasparri, Jason Luque, and Zachary F. Lerner. Proportional Joint-Moment Control for Instantaneously Adaptive Ankle Exoskeleton Assistance. *IEEE Transactions on Neural Systems and Rehabilitation Engineering*, 27(4):751–759, 2019.

[45]G. Orekhov, Y. Fang, J. Luque, and Z.F. Lerner. Ankle Exoskeleton Assistance Can Improve Over-Ground Walking Economy in Individuals with Cerebral Palsy. *IEEE Transactions on Neural Systems and Rehabilitation Engineering*, 2020.

[46]Greg Orekhov, Ying Fang, Chance F. Cuddeback, and Zachary F. Lerner. Usability and performance validation of an ultra-lightweight and versatile untethered robotic ankle exoskeleton. *Journal of NeuroEngineering and Rehabilitation*, 18(1):1–16, 2021.

[47]Ying Fang and Zachary F. Lerner. Feasibility of Augmenting Ankle Exoskeleton Walking Performance With Step Length Biofeedback in Individuals With Cerebral Palsy. *IEEE Transactions on Neural Systems and Rehabilitation Engineering*, 29:442–449, 2021.

[48]Benjamin C. Conner, Jason Luque, and Zachary F. Lerner. Adaptive Ankle Resistance from a Wearable Robotic Device to Improve Muscle Recruitment in Cerebral Palsy. *Annals of Biomedical Engineering*, 48(4):1309–1321, apr 2020.

[49]Benjamin C. Conner, Greg Orekhov, and Zachary Lerner. Ankle exoskeleton assistance increases six-minute walk test performance in cerebral palsy. *IEEE Open Journal of Engineering in Medicine and Biology*, PP(Table 1):1–1, 2021.

[50]Ying Fang, Greg Orekhov, and Zachary F Lerner. Improving the Energy Cost of Incline Walking and Stair Ascent With Ankle Exoskeleton Assistance in Cerebral Palsy. *IEEE Transactions on Biomedical Engineering*, 69(7):2143–2152, jul 2022.

[51]Ying Fang and Zachary F Lerner. Bilateral vs. Paretic-Limb-Only Ankle Exoskeleton Assistance for Improving Hemiparetic Gait: A Case Series. *IEEE Robotics and Automation Letters*, 7(2):1246–1253, apr 2022.

[52]Jesus de Miguel-Fernandez, Camille Pescatore, Alba Mesa-Garrido, Cindy Rikhof, Erik Prinsen, Josep M. Font-Llagunes, and Joan Lobo-Prat. Immediate Biomechanical Effects of Providing Adaptive Assistance With an Ankle Exoskeleton in Individuals After Stroke. *IEEE Robotics and Automation Letters*, 7(3):7574–7580, jul 2022.

[53]Jesus de Miguel-Fernandez, Alba Mesa-Garrido, Camille Pescatore, Cindy Rikhof, Joan Lobo-Prat, and Josep Maria Font- Llagunes. Relationship Between Ankle Assistive Torque and Biomechanical Gait Metrics in Individuals After Stroke. *preprint at TechRxiv*, pages 0–9, 2022.

[54]Bing Chen, Bin Zi, Bin Zhou, and Zhengyu Wang. Implementation of Robotic Ankle-Foot Orthosis with an Impedance-Based Assist-as-Needed Control Strategy. *Journal of Mechanisms and Robotics*, 14(5):1–12, 2022.

[55]Jungyoon Kim, Sungjae Hwang, Ryanghee Sohn, Younghee Lee, and Youngho Kim. Development of an active ankle foot orthosis to prevent foot drop and toe drag in hemiplegic patients: A preliminary study. *Applied Bionics and Biomechanics*, 8(3-4):377–384, 2011.

[56]Haisheng Xia, Junghan Kwon, Prabhat Pathak, Jooeun Ahn, Peter B. Shull, and Yong-Lae Park. Design of A Multi-Functional Soft Ankle Exoskeleton for Foot-Drop Prevention, Propulsion Assistance, and Inversion/Eversion Stabilization. In *2020 8th IEEE RAS/EMBS International Conference for Biomedical Robotics and Biomechatronics (BioRob)*, volume 2020-Novem, pages 118–123. IEEE, nov 2020.

[57]Daniel Gomez-Vargas, Maria J. Pinto-Betnal, Felipe Ballen-Moreno, Marcela Munera, and Carlos A. Cifuentes. Therapy with T-FLEX Ankle-Exoskeleton for Motor Recovery: A Case Study with a Stroke Survivor. *Proceedings of the IEEE RAS and EMBS International Conference on Biomedical Robotics and Biomechatronics*, 2020-Novem(August):491–496, 2020.

[58]Daniel Gomez-Vargas, Felipe Ballen-Moreno, Patricio Barria, Rolando Aguilar, José M. Azorín, Marcela Munera, and Carlos A. Cifuentes. The Actuation System of the Ankle Exoskeleton T-FLEX: First Use Experimental Validation in People with Stroke. *Brain Sciences*, 11(4):412, mar 2021.

[59]Jaehyun Bae, Stefano Marco Maria De Rossi, Kathleen O'Donnell, Kathryn L. Hendron, Louis N. Awad, Thiago R. Teles Dos Santos, Vanessa L. De Araujo, Ye Ding, Kenneth G. Holt, Terry D. Ellis, and Conor J. Walsh. A soft exosuit for patients with stroke: Feasibility study with a mobile off-board actuation unit. In *IEEE International Conference on Rehabilitation Robotics*, volume 2015-Septe, pages 11–14, 2015.

[60]Louis N. Awad, Jaehyun Bae, Pawel Kudzia, Andrew Long, Kathryn Hendron, Kenneth G. Holt, Kathleen O'Donnell, Terry D. Ellis, and Conor J. Walsh. Reducing Circumduction and Hip Hiking During Hemiparetic Walking Through Targeted Assistance of the Paretic Limb Using a Soft Robotic Exosuit. *American Journal of Physical Medicine & Rehabilitation*, 96(10):S157–S164, oct 2017.

[61]Louis N. Awad, Jaehyun Bae, Kathleen O'Donnell, Stefano Marco Maria De Rossi, Kathryn Hendron, Lizeth H. Sloot, Pawel Kudzia, Stephen Allen, Kenneth G. Holt, Terry D. Ellis, and Conor J. Walsh. A soft robotic exosuit improves walking in patients after stroke. *Science Translational Medicine*, 9(400):eaai9084, jul 2017.

[62]Jaehyun Bae, Louis N. Awad, A Long, K O'Donnell, Kathryn L. Hendron, Kenneth G. Holt, Terry D. Ellis, and Conor J. Walsh. Biomechanical mechanisms underlying exosuit-induced improvements in walking economy after stroke. *Journal of Experimental Biology*, 221(5), 2018.

[63]Christopher Siviy, Jaehyun Bae, Lauren Baker, Franchino Porciuncula, Teresa Baker, Terry D. Ellis, Louis N. Awad, and Conor James Walsh. Offline Assistance Optimization of a Soft Exosuit for Augmenting Ankle Power of Stroke Survivors During Walking. *IEEE Robotics and Automation Letters*, 5(2):828–835, apr 2020.

[64]Minhyung Lee, Jeonghun Kim, Seungyong Hyung, Jusuk Lee, Keehong Seo, Young Jin Park, Joonkee Cho, Byung Kwon Choi, Youngbo Shim, and Hyundo Choi. A Compact Ankle Exoskeleton with a Multiaxis Parallel Linkage Mechanism. *IEEE/ASME Transactions on Mechatronics*, 26(1):191–202, 2021.

[65]Luke M Mooney, Elliott J Rouse, and Hugh M Herr. Autonomous exoskeleton reduces metabolic cost of walking. In *2014 36th Annual International Conference of the IEEE Engineering in Medicine and Biology Society*, volume 11, pages 3065–3068. IEEE, aug 2014.

[66]Luke M Mooney, Rouse Elliot J, Hugh M Herr, Elliot J Rouze, and Hugh M Herr. Autonomous exoskeleton reduces metabolic cost of human walking during load carriage. *Journal of NeuroEngineering and Rehabilitation*, 11(1):80, aug 2014.

[67]Luke M. Mooney and Hugh M. Herr. Biomechanical walking mechanisms underlying the metabolic reduction caused by an autonomous exoskeleton. *Journal of NeuroEngineering and Rehabilitation*, 13(1):4, dec 2016.

[68]Xiangyu Peng, Yadrianna Acosta-Sojo, Man I. Wu, and Leia Stirling. Actuation Timing Perception of a Powered Ankle Exoskeleton and Its Associated Ankle Angle Changes during Walking. *IEEE Transactions on Neural Systems and Rehabilitation Engineering*, 30:869–877, 2022.

[69]Yadrianna Acosta-Sojo and Leia Stirling. Individuals differ in muscle activation patterns during early adaptation to a powered ankle exoskeleton. *Applied Ergonomics*, 98:103593, jan 2022.

[70]Sarah Gonzalez, Paul Stegall, Stephen M. Cain, Ho Chit Siu, and Leia Stirling. Assessment of a powered ankle exoskeleton on human stability and balance. *Applied Ergonomics*, 103:103768, sep 2022.

[71]M K Shepherd, D D Molinaro, G S Sawicki, and A J Young. Deep Learning Enables Exoboot Control to Augment Variable-Speed Walking. *IEEE Robotics and Automation Letters*, 7(2):3571–3577, 2022.

[72]Rachel W. Jackson and Steven H. Collins. An experimental comparison of the relative benefits of work and torque assistance in ankle exoskeletons. *Journal of Applied Physiology*, 119(5):541–557, sep 2015.

[73]Kirby Ann Witte, Juanjuan Zhang, Rachel W. Jackson, and Steven H. Collins. Design of two lightweight, high-bandwidth torque-controlled ankle exoskeletons. In *Proceedings - IEEE International Conference on Robotics and Automation*, volume June, pages 1223–1228, Dept. Mechanical Engineering, Carnegie Mellon University, 5000 Forbes Ave., Pittsburgh, PA, United States, 2015.

[74]Katherine M. Steele, Rachel W. Jackson, Benjamin R. Shuman, and Steven H. Collins. Muscle recruitment and coordination with an ankle exoskeleton. *Journal of Biomechanics*, 59:50–58, jul 2017.

[75]Juanjuan Zhang, Pieter Fiers, Kirby A Witte, Rachel W Jackson, Katherine L Poggensee, Christopher G Atkeson, and Steven H Collins. Human-in-the-loop optimization of exoskeleton assistance during walking. *Science*, 356(6344):1280–1284, jun 2017.

[76]Rachel W Jackson and Steven H Collins. Heuristic-Based Ankle Exoskeleton Control for Co-Adaptive Assistance of Human Locomotion. *IEEE Transactions on Neural Systems and Rehabilitation Engineering*, 27(10):2059–2069, oct 2019.

[77]Patrick Slade, Mykel J Kochenderfer, Scott L Delp, and Steven H Collins. Personalizing exoskeleton assistance while walking in the real world. *Nature*, 610(7931):277–282, oct 2022.

[78]Yacine Bougrinat, Sofiane Achiche, and Maxime Raison. Design and development of a lightweight ankle exoskeleton for human walking augmentation. *Mechatronics*, 64(March 2018):102297, 2019.

[79]Wietse van Dijk, Cory Meijneke, and Herman van der Kooij. Evaluation of the Achilles Ankle Exoskeleton. *IEEE Transactions on Neural Systems and Rehabilitation Engineering*, 25(2):151–160, feb 2017.

[80]Florin Dzeladini, Amy R. Wu, Daniel Renjewski, Arash Arami, Etienne Burdet, Edwin van Asseldonk, Herman van der Kooij, and Auke J. Ijspeert. Effects of a neuromuscular controller on a powered ankle exoskeleton during human walking. In *2016 6th IEEE International Conference on Biomedical Robotics and Biomechatronics (BioRob)*, volume 2016-July, pages 617–622. IEEE, jun 2016.

[81]F. Tamburella, N. L. Tagliamonte, I. Pisotta, M. Masciullo, M. Arquilla, E. H.F. Van Asseldonk, H. Van Der Kooij, A. R. Wu, F. Dzeladini, A. J. Ijspeert, and M. Molinari. Neuromuscular Controller Embedded in a Powered Ankle Exoskeleton: Effects on Gait, Clinical Features and Subjective Perspective of Incomplete Spinal Cord Injured Subjects. *IEEE Transactions on Neural Systems and Rehabilitation Engineering*, 28(5):1157–1167, 2020.

[82]Cor Meijneke, Wietse Van Dijk, and Herman Van Der Kooij. Achilles: An autonomous lightweight ankle exoskeleton to provide push-off power. *Proceedings of the IEEE RAS and EMBS International Conference on Biomedical Robotics and Biomechatronics*, pages 918–923, 2014.

[83]Keith E Gordon and Daniel P. Ferris. Learning to walk with a robotic ankle exoskeleton. *Journal of Biomechanics*, 40(12):2636–2644, jan 2007.

[84]Catherine R. Kinnaird and Daniel P. Ferris. Medial gastrocnemius myoelectric control of a robotic ankle exoskeleton. *IEEE Transactions on Neural Systems and Rehabilitation Engineering*, 17(1):31–37, 2009.

[85]Stephen M. Cain, Keith E. Gordon, and Daniel P. Ferris. Locomotor adaptation to a powered ankle-foot orthosis depends on control method. *Journal of NeuroEngineering and Rehabilitation*, 4:1–13, 2007.

[86]Pei-Chun Kao, Cara L. Lewis, and Daniel P. Ferris. Invariant ankle moment patterns when walking with and without a robotic ankle exoskeleton. *Journal of Biomechanics*, 43(2):203–209, jan 2010.

[87]Gregory S Sawicki and Daniel P Ferris. Mechanics and energetics of level walking with powered ankle exoskeletons. *The Journal of experimental biology*, 211(Pt 9):1402–1413, may 2008.

[88]Jeffrey R Koller, Daniel A Jacobs, Daniel P Ferris, and C. David Remy. Learning to walk with an adaptive gain proportional myoelectric controller for a robotic ankle exoskeleton. *Journal of neuroengineering and rehabilitation*, 12(1):97, nov 2015.

[89]Jeffrey R. Koller, C. David Remy, and Daniel P. Ferris. Comparing neural control and mechanically intrinsic control of powered ankle exoskeletons. *IEEE International Conference on Rehabilitation Robotics*, pages 294–299, 2017.

[90]Cristina Bayón, Arvid Q L Keemink, Michelle van Mierlo, Wolfgang Rampeltshammer, Herman van der Kooij, and Edwin H F van Asseldonk. Cooperative ankle-exoskeleton control can reduce effort to recover balance after unexpected disturbances during walking. *Journal of NeuroEngineering and Rehabilitation*, 19(1):21, dec 2022.

[91]Guillaume Durandau, Wolfgang F Rampeltshammer, Herman van der Kooij, and Massimo Sartori. Neuromechanical Model-Based Adaptive Control of Bilateral Ankle Exoskeletons: Biological Joint Torque and Electromyogram Reduction Across Walking Conditions. *IEEE Transactions on Robotics*, 38(3):1380–1394, jun 2022.

[92]C Meijneke, G van Oort, V Sluiter, E van Asseldonk, N L Tagliamonte, F Tamburella, I Pisotta, M Masciullo, M Arquilla, M Molinari, A R Wu, F Dzeladini, A J Ijspeert, and H van der Kooij. Symbitron Exoskeleton: Design, Control, and Evaluation of a Modular Exoskeleton for Incomplete and Complete Spinal Cord Injured Individuals. *IEEE transactions on neural systems and rehabilitation engineering : a publication of the IEEE Engineering in Medicine and Biology Society*, 29:330–339, 2021.

[93]K. Alex Shorter, Gza F. Kogler, Eric Loth, William K. Durfee, and Elizabeth T. Hsiao-Wecksler. A portable powered ankle-foot orthosis for rehabilitation. *The Journal of Rehabilitation Research and Development*, 48(4):459, 2011.

[94]David Yifan Li, Aaron Becker, Kenneth Alex Shorter, Timothy Bretl, and Elizabeth T. Hsiao-Wecksler. Estimating system state during human walking with a powered ankle-foot orthosis. *IEEE/ASME Transactions on Mechatronics*, 16(5):835–844, 2011.

[95]A. H. Weerasingha, A. D.K.H. Pragnathilaka, W. P.K. Withanage, R. K.P.S. Ranaweera, and R. A.R.C. Gopura. C-JAE: 3 DOF robotic ankle exoskeleton with compatible joint axes. *MERCon 2018 - 4th International Multidisciplinary Moratuwa Engineering Research Conference*, pages 270–275, 2018.

[96]Chao Zhang, Yanhe Zhu, Jizhuang Fan, Jie Zhao, and Hongying Yu. Design of a quasi-passive 3 DOFs ankle-foot wearable rehabilitation orthosis. *Bio-Medical Materials and Engineering*, 26:S647–S654, 2015.

[97]S Galle, P Malcolm, W Derave, and D De Clercq. Adaptation to walking with an exoskeleton that assists ankle extension. *Gait & Posture*, 38(3):495–499, jul 2013.

[98]Samuel Galle, Philippe Malcolm, Wim Derave, and Dirk De Clercq. Enhancing performance during inclined loaded walking with a powered ankle–foot exoskeleton. *European Journal of Applied Physiology*, 114(11):2341–2351, nov 2014.

[99]S. Galle, P. Malcolm, W. Derave, and D. De Clercq. Uphill walking with a simple exoskeleton: Plantarflexion assistance leads to proximal adaptations. *Gait and Posture*, 41(1):246–251, 2015.

[100]Samuel Galle, Philippe Malcolm, Steven Hartley Collins, and Dirk De Clercq. Reducing the metabolic cost of walking with an ankle exoskeleton: interaction between actuation timing and power. *Journal of NeuroEngineering and Rehabilitation*, 14(1):1–16, 2017.

[101]Philippe Malcolm, Samuel Galle, Pieter Van Den Berghe, and Dirk De Clercq. Exoskeleton assistance symmetry matters: Unilateral assistance reduces metabolic cost, but relatively less than bilateral assistance. *Journal of NeuroEngineering and Rehabilitation*, 15(1), 2018.

[102]Weiguang Huo, Victor Arnez-Paniagua, Guangzheng Ding, Yacine Amirat, and Samer Mohammed. Adaptive Proxy-Based Controller of an Active Ankle Foot Orthosis to Assist Lower Limb Movements of Paretic Patients. *Robotica*, 37(12):2147–2164, dec 2019.

[103]Ho Seon Choi, Chang Hee Lee, and Yoon SuBaek. Design and Validation of a Two-Degree-of-Freedom Powered Ankle-Foot Orthosis with Two Pneumatic Artificial Muscles. *Mechatronics*, 72, 2020.
